# Pulmonary intimal sarcoma involving the pulmonary valve and right ventricular outflow tract: A case report and literature review

**DOI:** 10.1097/MD.0000000000018813

**Published:** 2020-01-17

**Authors:** Rui Xu, Yixuan Zhao, Xiaosen Xu, Shuang Liu, Chenyu Hu, Dongmei Lv, Huiying Wu

**Affiliations:** a Department of Ultrasonography, Second Hospital of Jilin University; b Department of Ultrasonography, Changchun Traditional Chinese, Medicine Hospital, Changchun, Jilin, China.

**Keywords:** diagnosis, pulmonary artery, pulmonary thromboembolism, radiological imaging, sarcoma

## Abstract

**Introduction:**

Pulmonary artery intimal sarcoma (PAIS) is a rare and highly aggressive tumor, and approximately 80% of pulmonary cases occur in the pulmonary trunk. We report herein a case of retrograde extension of the sarcoma to the pulmonary valve and right ventricle, which is an uncommon manifestation of this lethal tumor.

**Patient concerns:**

A 41-year-old woman was initially diagnosed with pulmonary thromboembolism (PTE) and transferred to our hospital.

**Diagnosis:**

Computed tomographic pulmonary angiography (CTPA) showed that there are low-density filling defects in both pulmonary arteries, and the patient was diagnosed with PTE. However, the ultrasonographers considered that the lesion is a space-occupying type that involves the right ventricular outflow tract and pulmonary valve instead of PTE. Postoperative pathology confirmed the diagnosis of PAIS.

**Interventions:**

The patient underwent resection of pulmonary artery sarcoma and endarterectomy.

**Outcomes:**

During the follow-up via telephone 1 month after discharge, the patient reported to have been feeling well.

**Conclusion:**

Owing to the rarity of the disease and its non-specific clinical manifestations, approximately half of the PAIS cases are misdiagnosed or have a delayed diagnosis. Thus, improving our understanding of the disease and facilitating its early diagnosis are essential.

## 1 Introduction

Pulmonary artery intimal sarcoma (PAIS) is very rare and arises from primitive pluripotent mesenchymal cells with multi-directional differentiation capacity.^[1]^ Its incidences is only 0.001% to 0.03% and undifferentiated sarcoma (34%) is the most common pathological type.^[2]^ Owing to the low incidence of the disease, we still do not understand its etiology and risk factors. It is often misdiagnosed because its clinical and imaging manifestations are similar to pulmonary thromboembolism (PTE). At present, the diagnosis of pulmonary artery (PA) endometrial sarcoma is based on the results of the autopsy and postoperative histopathology is considered the “golden standard”.^[3]^ Given its rapid growth, it can result in right heart failure and pulmonary obstruction in a short time, thus, early diagnosis and surgical resection are the key to its treatment.

We report herein a rare case of retrograde extension of the sarcoma to the pulmonary valve and right ventricle.

## 2 Case report

**Figure 1:**
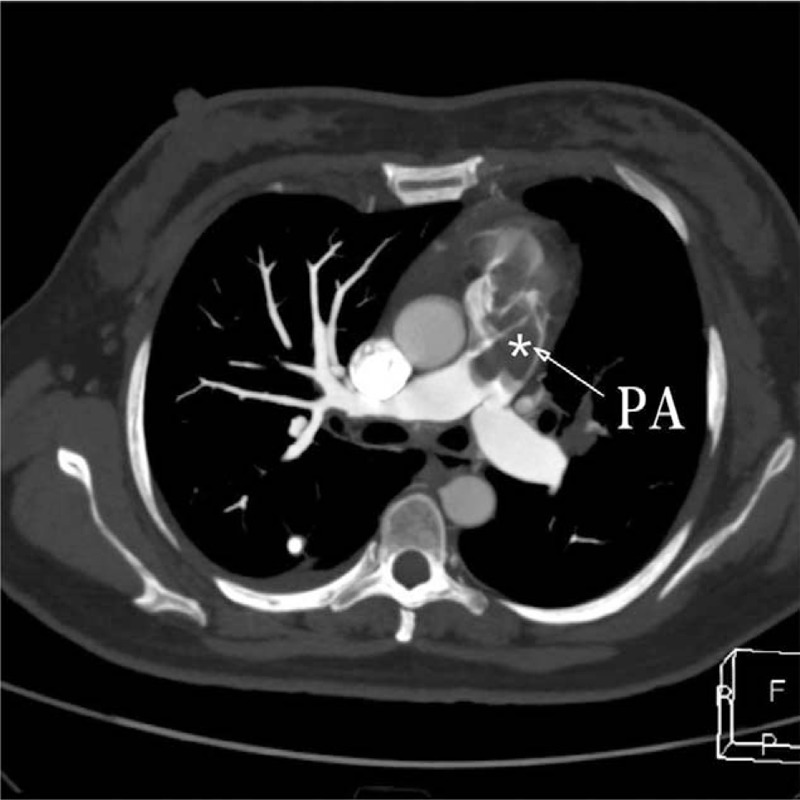
Pulmonary CTA showed that there are low density filling defects (asterisks) in the pulmonary arteries. PA = pulmonary artery.

**Figure 2:**
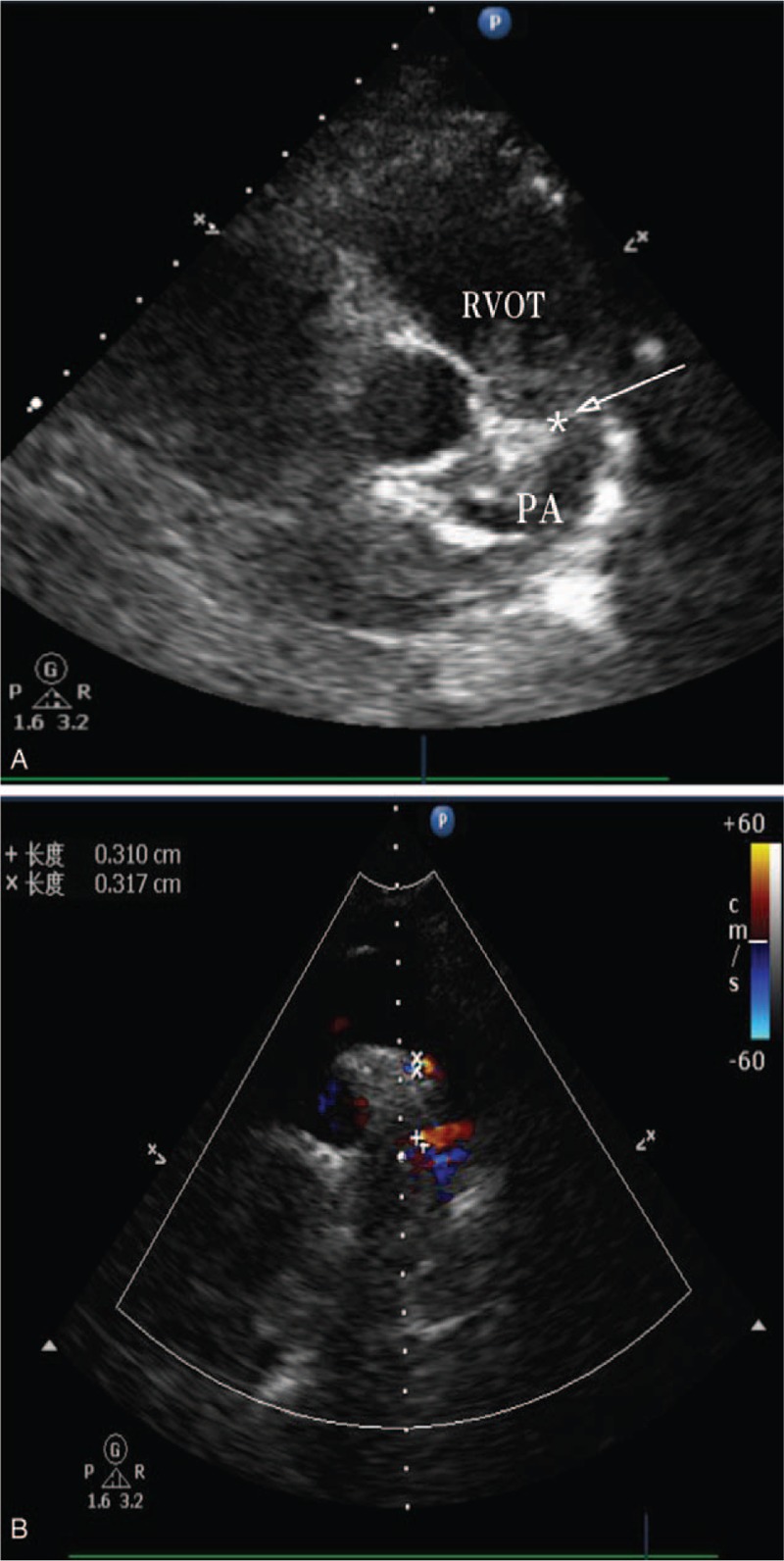
A: Transthoracic echocardiogram (parasternal short-axis view)shows the hypoechoic mass (asterisks)in the main pulmonary artery, extending down to the right ventricular outflow tract. RVOT=right ventricular outflow tract. B: The Color Doppler imaging demonstrates the presence of blood flow signals (asterisks) between the aorta and the pulmonary artery.

A 41-year-old woman who complained of chest tightness after performing an activity, presented with aggravation of symptoms in our hospital. She was diagnosed with pulmonary embolism in a different hospital, prior to her transfer in our institution. The patient occasionally had cough, expectoration, blood in the sputum, and nocturnal sweating, but she had no edema in the lower extremities, fever, smoking history, and related genetic history in the family. There were two transient syncope episodes in the course of the disease. Blood oxygen saturation was 72% and carbohydrate antigen 125(CA125) was 230.2 U/ml at admission. Other physical examinations and laboratory data were unremarkable. Computed tomographic pulmonary angiography (CTPA) showed that there are low-density filling defects in both PAs, and then the patient was diagnosed with PTE (Fig. 1). However, venous thrombosis could not be found in her lower extremities. Echocardiography showed at the proximal end of the main PA and its left and right branches, a hyperechoic mass of approximately 13 × 13 mm in size, which appeared to have invaded the pulmonary valve, extending down to the right ventricular outflow tract (Fig. 2A). It also showed an enlargement of the right heart, severe tricuspid regurgitation and pulmonary hypertension. Moreover, the lateral pulmonary branch of the body could be seen to form (Fig. 2B). Thus, ultrasonographers considered that the mass is a space-occupying lesion involving the right ventricular outflow tract and pulmonary valve instead of PTE.

**Figure 3:**
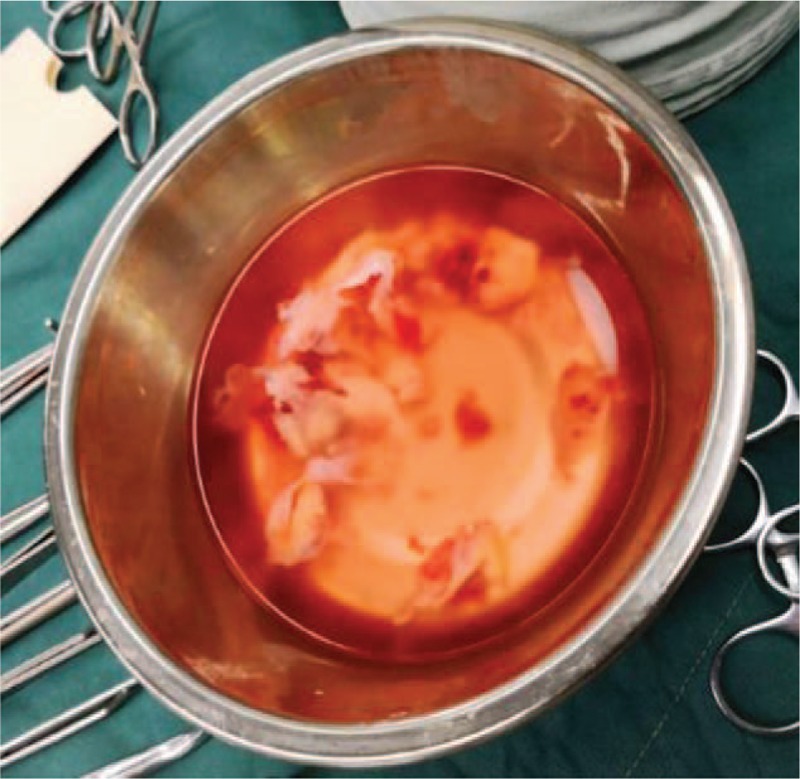
This is the tumor mass and intima that we took out of the pulmonary artery during the surgery.

**Figure 4:**
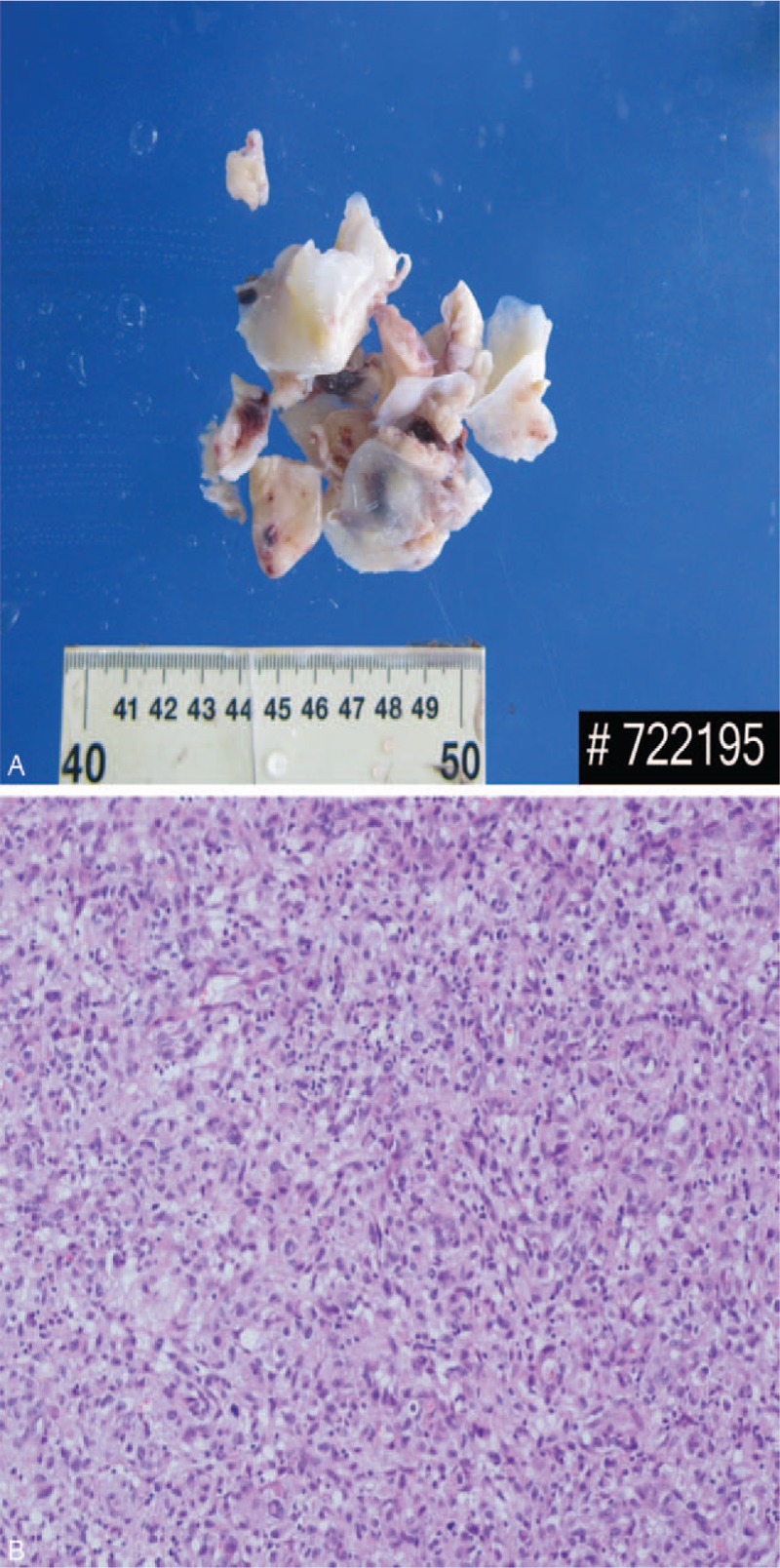
A: Partial stripping of the intima and sarcoma of the pulmonary artery. B: The lesion morphology showed polypoid changes, local regional cell proliferation, with atypia and sarcomatoid changes, consistent with high-grade pleomorphic undifferentiated sarcoma. Hematoxylin-eosin staining, original magnification.

Based on these diagnoses and the patient's clinical manifestations, emergency surgery is required. Intraoperatively, the ratio of the aorta to the main PA was found to be 1: 1.3. In the exploration of the main PA under the support of extracorporeal circulation, a large number of polyp like substances, which are irregular in shape, invaded the pulmonary valve, extending down to the septal side of the right ventricular outflow tract, and involving the far end of the left and right PAs. We performed a partial pulmonary endarterectomy and the sarcomatoid material was taken out from the PA (Fig. 3). The specimens (Fig. 4A)were submitted for intraoperative pathological examination, and the mass was diagnosed as an endometrial sarcoma of the PA, the histologic type is high-grade pleomorphic undifferentiated sarcoma (Fig. 4B). We then performed partial pulmonary valve resection and repair of the pulmonary trunk with a pericardium patch. Afterwards, tricuspidplasty was also conducted.

Postoperative echocardiography showed that the PA pressure was significantly reduced and the patient's condition was improved. The immunological histological chemistry (IHC) results were as follows: ER(−), PR(+), CD31(−), CD34(−), DOG(−), SMA(+), Desmin(+), H- Caldesmon(+), EMA(-), CK(AE1/AE3)(+), Vimentin(+), S-100(−), K167(30%), LCA(−), ALK(-), CD30(−), CD17(−), DOG-1(−), WT19(−)and calretinin (−). The patient stayed in the intensive care unit (ICU) for 3 days and transferred back to the general ward. However, she refused to undergo radiation and chemotherapy. During the follow-up via telephone 6 month after the operation, the patient reported that she has been feeling well.

## 3 Discussion

Intimal sarcomas are most commonly found in the pulmonary arteries and aorta, and pulmonary cases are almost twice as common as aortic cases.^[4]^ Approximately, 400 cases of PAIS have been reported since Mandelstamm first reported the disease, after he detected it during an autopsy in 1923.^[5,6]^ The average age at diagnosis is 49.3 years with a wide age range of 13 to 81 years.^[7]^ No significant difference in the sex ratio has been found until now, possibly because of the difficulty in obtaining diagnostic material and general unfamiliarity with the entity. Most of the PA tumors occur in the pulmonary trunk and bifurcation. A previous report demonstrated that PAIS had an intraluminal polypoid growth pattern macroscopically.^[8]^ At present, Kim et al^[9]^ introduced a new point of view based on computed tomography(CT) images. They believe that PAIS has different growth stages. At first, polypoid tumor foci of PAIS form in the intimal layer of the PA first; these subsequently extend along the vessel, forming a cauliflower-like mass or diffuse/focal wall thickening; Finally, the tumors become compact and occupy almost the entire PA lumen.^[9]^ Typically, intimal sarcomas of the pulmonary artery show histological features of undifferentiated/unclassified sarcoma with focal or diffuse myxoid changes.^[4]^ Microscopically, they show multifocal gelatinous, polypoidal nodules or plaques, comprising spindled or epithelioid cells with marked nuclear atypia.^[10]^ The morphology of differentiated sarcoma can be manifested as myofibroblastoma, malignant fibrohistiocytoma, angiosarcoma, leiomyosarcoma, rhabdomyosarcoma, myxoid chondrosarcoma, and so on. Therefore, a variety of tumor components can be observed in the differentiated sarcoma, among which angiosarcoma is the most common one.^[11]^

The onset of PAIS is occult. Its clinical symptoms and signs are not specific. Moreover the early symptoms may be clinical manifestations of pulmonary embolism, such as chest pain, hemoptysis, sexual dyspnea, and with the increase of tumor, it can cause stenosis of the lumen, thereby increasing the pulmonary artery pressure. The corresponding clinical symptoms, such as cyanosis, edema, jugular vein rage, hepatomegaly and splenomegaly, may occur in the late stage of pulmonary circulation obstruction, which may result in distant metastasis.^[12]^ Physical examination showed pulmonary hypertension and hyperactivity of the second heart sounds in the pulmonary region due to the high right ventricular load, and systemic congestion. The most common manifestation is a systolic murmur in the pulmonary area. Xin et al^[13]^ analyzed patients’ data and found elevated serum inflammatory markers, including an elevated erythrocyte sedimentation rate, lactate dehydrogenase level and C-reactive protein level. Serum levels of carcinoembryonic antigen were within the reference range in all of the patients.^[13]^ These clinical features and laboratory results have no obvious specificity. Now, there are some studies showing that the in situ hybridization results for MDM2 amplification were helpful in determining the accurate diagnosis.^[10]^ The diagnostic findings in PAIS are over expression of MDM2 and gains and amplification of the 12q13–14 region.^[14]^

Until recently, considerable advances have been made in the use of imaging technologies for the diagnosis of PAIS before surgery. We performed a comparative analysis to each kind of image examination in the following texts. Transthoracic echocardiography's (TTE) main function is to provide the relevant information of the pulmonary valve and the right heart, such as whether the pulmonary artery has been eroded and whether the right heart is dysfunctional. It also can be used to evaluate PAIS by location, morphology, relationship with surrounding tissues, hemodynamics and other aspects. However, it requires ultrasonographers to have high technical and clinical experience. Moreover, the effects of the PA lesions on other cardiac structures and pulmonary physiology were not recognized.^[13]^ Based on the abovementioned situation, although we cannot rely on it to make a clear diagnosis due to the limitations of TTE, a series of image prompt messages brought by it are still indispensable. Diagnosis of pulmonary artery sarcoma (PAS) by endobronchial ultrasound-guided transbronchial needle aspiration has also been reported previously.^[15]^

CTPA is often our preferred method of examination, although the findings of CTPA for PAS and PTE are similar with respect to the filling defects in the pulmonary trunk, which is the main reason why we often misdiagnose them.^[16,17]^ A review of literature helps in differentiating these diseases from each other. PAS lesions are continuous, with rounded, bulging, or lobulated surfaces that project in the direction of blood flow, they develop as the tumor tissue accumulates, expands and invades surrounding tissues.^[13]^ Pulmonary emboli appears as cup-like structures in CT images when were viewed against the blood flow. This may be the result of friction caused by blood flow at the surface clot, which is undergoing dissolution by the fibrinolytic system of the blood.^[17]^ The presence of extraluminal tumor extension and distant metastases is a reliable sign in the differential diagnosis, but these are manifestations of advanced cancer of intimal sarcoma.^[18]^ Although both show enhancement on the CTPA, intimal sarcoma tends to present as a unilateral, central, lobulated, PA filling defect which increases in size along with the increasing diameter of the PAs and has acute angles with the vessel wall, in contrast to the PTE.^[18–22]^ Moreover, intimal sarcoma shows heterogeneous densities due to areas of necrosis, hemorrhage, and ossification within the mass.^[23]^ Therefore, the detection of morphological differences in lesions during CTPA is critical for distinguishing PAS from PTE.^[13]^ The positron emission tomography (PET)-CT was used to distinguish PTE on the basis of the increased radio pharmaceutical uptake of tumors. Moreover, it is also sensitive to distant metastases. Now, magnetic resonance imaging (MRI) plays an increasingly important role in the diagnosis of PAIS. The excellent spatial and tissue resolution in MRI is advantageous for the diagnosis of PAS and it allows for tissue characterization, providing insight into the tumor type.^[13,24]^ Cine MRI can be used to observe the movement of a PAS tumor in response to the changes in blood flow between pulmonary diastole and systole.^[25]^ The MRI signals of a PTE vary across the different stages of thrombosis, and can appear as high signals in both T1W and T2W scans.^[13]^ After MRI enhanced scanning, PAS showed a heterogeneous enhancement, whereas PE did not. However, many patients with PAS have difficulty, holding their breath long enough to allow MRI scanning, which limits the use of MRI for the diagnosis of PAS.^[13]^

PAIS is a malignant tumor that progresses rapidly. If the intervention is delayed, the prognosis is extremely poor and the recurrence rate is high. At present, the main treatment for PAS is resection of sarcoma. Compared with isolated tumor resection, pulmonary endarterectomy seemed to yield a better survival rate.^[26]^ Even after complete surgery, the prognosis of PAIS is poor because of frequent relapses, more often local than distant.^[27]^ Postoperative chemotherapy and/or radiotherapy have been shown to improve the efficacy of the available treatments for PAS, but there are no guidelines for this.^[28,29]^ Anthracyc-lines, especially doxorubicin, are a standard drug in the treatment of soft tissue sarcomas.^[30]^ It is reported that combining cisplatin and vinorelbine as the 2nd therapeutic line, which was better tolerated and led to a remarkable partial response.^[27]^ Molecular evidences have shown a potential effect of tyrosine kinase inhibitors on intimal sarcoma.^[31]^ However, the use of MDM2 small-molecule antagonists to block the interaction between p53 and MDM2, restore the p53 pathway, and inhibit tumor cell proliferation has been reported.^[32]^

Even if treated, the postoperative survival rates is still poor, with a median overall survival ≤18 months.^[33]^ In previous reports, the mean survival in untreated patients was approximately 1.5 months, and even in cases when surgical resection of the tumor was performed, more than half of the patients died within 1 year after surgery.^[34,35]^ Recently, Bandyopadhyay et al reviewed a total of 391 cases previously reported in the English language literature and revealed that patients who underwent complete surgeries had significantly improved survival compared to those who underwent partial resection.^[36]^ The specific factors affecting prognosis are not clear, including:

1. Whether the tumor can be completely resected, whether the tumor has local recurrence and whether there is distant metastasis
2. Pathological types, such as smooth muscle sarcoma has a relatively good prognosis, whereas rhabdomyosar-coma has the worst prognosis and well-differentiated muscle fibers retinoblastosarcoma may have a good prognosis.^[37]^
3. Age of onset, patients aged <40 years have usually a better prognosis
4. Postoperative chemoradiotherapy can improve the prognosis.

PAS is often misdiagnosed because of the same hemodynamic changes and clinical manifestations with chronic pulmonary embolism. We have reviewed the related literature, summarized and analyzed the differences in the following aspects

1. Medical history: Patients with PAIS have a long course of disease, which is manifested by recent aggravation without a history of bed rest, surgery, fracture, and so on. PE patients usually have a sudden onset and have a history of corresponding thrombosis, with more acute and rapid clinical manifestations.
2. Clinical manifestation: PAIS patients with insidious onset, have clinical symptoms and signs without specificity. In addition to the symptoms, including dyspnea, syncope, palpitations, chest pain and hemoptysis, the constitutional symptoms, such as fever fatigue and weight loss were most prominent in our patients.^[13]^ This information deserves our utmost attention.
3. Laboratory examination: In patients with PAIS, the level of d-dimer is usually within the normal range, whereas serum inflammatory markers are elevated. Therefore, anticoagulation therapy is ineffective. Moreover, arterial blood gas usually indicates hypoxemia and hypocapnia, which are related to the imbalance of ventilation/blood flow ratio.
4. Imaging examination:
  a. TTE is mainly used for right ventricular outflow tract pulmonary artery and its branch lesions to show the relationship with the surrounding tissues and whether there is an invasion. PAIS has an uneven internal echo and certain blood flow signals, and the ’oscillation phenomenon’ is present when it invades the pulmonary valve.
  b. MRI or CT: Pulmonary sarcomas are characterized by mass filling shadows in the main PA, the left and right PAs and even right ventricular outflow tract, show an uneven density, irregular boundary, lobulated, nodular or septal phenomena. CT can be enhanced to show pulmonary sarcomas with erosion-like changes in the walls of the PAs, and even the extraluminal infiltration can be seen.
  c. PET-CT: In brief, PAIS was positive for FDG uptake and negative for thrombus like malignant tumors;on the other hand, the mean SUV value of PAIS was (7.63 ± 2.21) and the thrombus was (2.31 ± 0.41).^[38–40]^

In conclusion, high-risk factors and specific molecular markers need further study on the tissue origin of PAS. Our data could contribute to the improvement of the understanding of the disease, symptoms of patients, and short-term survival.

## Author contributions

**Conceptualization:** Shuang Liu.

**Writing – original draft:** Rui Xu, Chenyu Hu.

**Writing – review & editing:** DongMei Lv, HuiYing Wu.
